# Predictive factors for intraoperative blood loss in surgery for adolescent idiopathic scoliosis

**DOI:** 10.1186/s12891-021-04104-z

**Published:** 2021-02-26

**Authors:** Chris Yuk Kwan Tang, Vijay H. D. Kamath, Prudence Wing Hang Cheung, Jason Pui Yin Cheung

**Affiliations:** Department of Orthopaedics and Traumatology, The University of Hong Kong, Pokfulam, Hong Kong SAR, China

**Keywords:** Adolescent idiopathic scoliosis, Blood loss, Predictive factors

## Abstract

**Background:**

Adolescent idiopathic scoliosis (AIS) is a common spinal deformity. Posterior spinal fusion remains an important surgical treatment for AIS. This study aims to determine the predictive factors for intraoperative blood loss in AIS surgery.

**Methods:**

Patients who had undergone posterior spinal fusion for adolescent idiopathic scoliosis in a single university hospital were reviewed over a 7-year period. Predictive factors for intra-operative blood loss were studied by multivariate analysis to derive a regression model. Receiver operating characteristic analysis was performed to determine the cut-off values of factors contributing to significant intraoperative blood loss (≥500 ml).

**Results:**

Two hundred and twelve patients were included. Intraoperative blood loss was found to be correlated with gender (*r*_*s*_ = 0.30 (0.17–0.43)), preoperative hemoglobin level (*r*_*s*_ = 0.20 (0.04–0.31)), preoperative Cobb angle (*r*_*s*_ = 0.20 (0.02–0.29)), number of fused levels (*r*_*s*_ = 0.46 (0.34–0.58)), operation duration (*r*_*s*_ = 0.65 (0.54–0.75)), number of anchors (*r*_*s*_ = 0.47 (0.35–0.59)), and *p*-value ranged from < 0.001 to < 0.05. Significant intraoperative blood loss was influenced by the male gender, operation duration greater than 257.5 min and more than 10 anchors used.

**Conclusions:**

Male gender, increased operation duration and higher number of anchors predicted higher intra-operative blood loss.

## Introduction

Posterior spinal fusion is a common surgical option in the management of adolescent idiopathic scoliosis (AIS). Minimising blood loss is one important goal in AIS surgery. Blood loss in AIS surgery can occur intra-operatively or post-operatively. Excessive intra-operative blood loss leads to blood transfusion, which has been shown to increase risk of surgical site infection in spinal surgery [1], resulting in morbidity to the patients, increased hospital stay and increased costs to treatment. Also, increased blood loss had been shown to increase the chance of non-neurologic complications in AIS surgery, namely respiratory complications, wound infections, wound hematoma, seroma, and dehiscence [2]. On the other hand, identifying the group of patients that will have a higher chance of significant intra-operative blood loss is equally important, since this facilitates blood-salvaging preparation and counselling of patients and their families before the operation. Moreover, precautions should be employed in order to minimise intra-operative blood loss in AIS surgery.

The purpose of this study was to review the outcome of the patients who underwent surgery for AIS in terms predictive factors for intra-operative blood loss.

## Materials and methods

The records of patients with AIS who had undergone posterior spinal fusion in a single university hospital over a 7-year period were reviewed. This study was ethically approved by a local institutional review board. All patients had minimum 2-year postoperative follow-up. Exclusion criteria were patients who had undergone spinal osteotomies as part of the surgical procedure, bleeding disorders and those who were on anticoagulation therapy. Patients who underwent spinal osteotomies were excluded because the blood loss was more extensive in this group of patients.

The clinical records were scrutinized for patients’ age, gender, body mass index (BMI), curve type according to the Lenke Classification, major curve Cobb angle, curve flexibility (preoperative Cobb angle – Cobb angle in fulcrum bending radiograph / preoperative Cobb angle), amount of curve correction, duration of surgery, number of levels fused, and number and type of anchor points. Wound complications were charted according to Southampton Wound Assessment Scale [3] (Grade 0: normal healing; Grade I: normal healing with mild bruising or hematoma; Grade II: erythema plus other signs of inflammation; Grade III: clear or hemoserous discharge; Grade IV: pus; Grade V: deep or severe wound infection).

All patients were anaesthetised by the same anesthetic team. Hypotensive anaesthesia was performed for all patients to keep 80% of their usual mean arterial pressure. None of the patients used any tranexemic acid. Three independent spine specialists in the same surgical team were involved in all operations at a university unit. All patients underwent a similar surgical procedure and received antibiotic prophylaxis with cephazolin (1st generation cephalosporin) on induction of anesthesia. At the onset of the surgery, autogenous iliac-crest bone graft was harvested from the posterior iliac crest. After the harvest, hemostasis was achieved with bone wax, and the iliac wound was closed in layers with or without the insertion of a suction drain, which was surgeon-dependent. The graft was placed aside and a standard midline sub-periosteal dissection up to the tips of the transverse processes of the levels to be fused was performed. Alternate-level pedicle screws, hooks or wires were inserted and attached to contoured rods. Additional strategic anchors were inserted at the apex for stiffer curves. In order to decrease blood loss, bone wax or finger pressure was used to seal the pedicle track after confirming integrity and screw insertion. Also, the suction catheter tip was away from the screw entry point to limit suction of the oozing blood. Curve correction was then obtained by concave rod rotation and segmental concave distraction, followed by convex rod insertion and segmental compression. Intraoperative radiographs were obtained to assess the fusion mass and any adjustments were performed [4]. The two rods were connected by two crosslinks placed at the cranial and caudal aspects of the construct. Inferior articular facets of the spine were removed and facet fusion was performed with autogenous iliac crest bone graft. The lamina and transverse processes were decorticated with the remaining bone graft laid in bed. No topical antibiotics was placed in the wound. Two surgeons closed the deep fascia simultaneously in a water-tight manner with continuous 1/0 vicryl sutures (polyglactin, Ethicon Sommerville, New Jersey) to reduce blood loss. A closed suction system drain (Exudrain™) was placed in the subcutaneous layer and closure of the subcutaneous tissue was done with continuous 2/0 vicryl sutures (polyglactin, Ethicon). The incision was then closed with 3/0 biosyn (polypropylene, Ethicon) sutures in a running subcuticular fashion with re-enforcement with Steri-strips (3 M, St Pauls, Minnesota). The drain was anchored to the skin to prevent inadvertent pull out. No hemostatic agents including tranexamic acid were used intra- or postoperatively. Intraoperative blood loss was estimated by the total amount of intra-operative suction from the wound minus the amount of irrigation used.

**Fig. 1:**
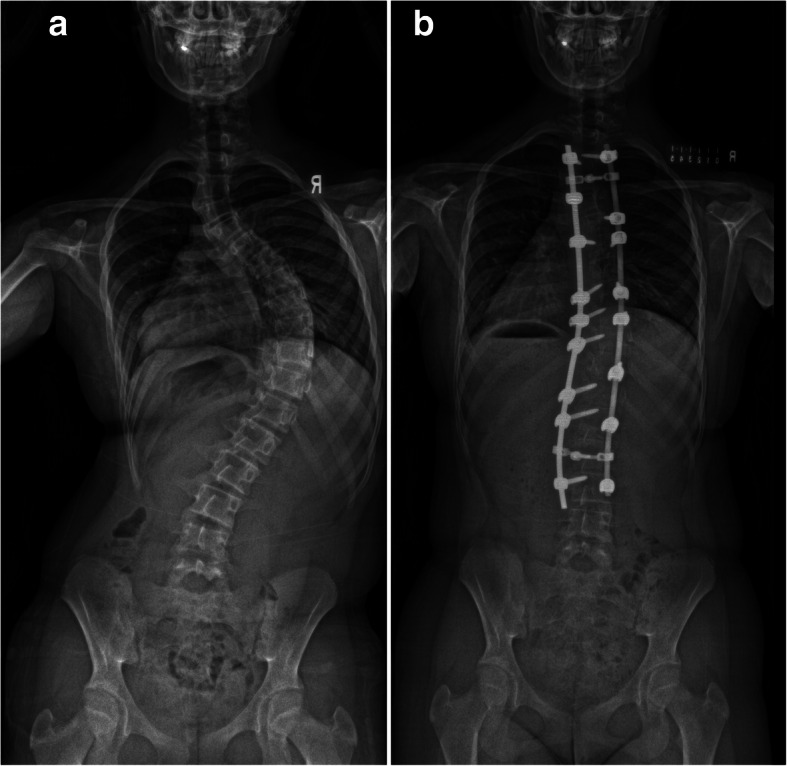
Pre-operative (**a**) and post-operative (**b**) radiographs of a 12-year-old girl with T2-L3 posterior spinal fusion for the Lenke 1 curve. Total operative time was 320 min with intra-operative blood loss of 900 ml

A standardised postoperative care protocol was followed at the institution. Patients were allowed to mobilize immediately. The drains were removed when the drain collection was less than 30 ml over a 24-h period. Patients were encouraged to sit out of bed in a chair and ambulate as pain allowed under the supervision of a physiotherapist. The patients were discharged when they were able to ambulant independently. A patient with pre-operative and post-operative radiographs was shown as an example (Fig. 1).

### Statistical analysis

Descriptive statistics were reported as mean values ± standard deviation (SD). Normality of data was tested with Shapiro-Wilk test. Univariate analysis was conducted through Spearman’s (*r*_*s*_) and point-biserial (*r*_pb_) correlation tests to investigate the relationships between the following factors and intraoperative blood loss: number of levels fused, number of anchor points, duration of surgery, BMI, age, preoperative haemoglobin (g/dL), duration of surgery, Cobb angle, spinal flexibility and preoperative albumin level (nutritional status). Any independent variables that reached a statistical significance of < 0.05 were then included in the multivariate models. Linearity test was performed, and multiple regression was used to derive a regression equation with predictors being identified.

Based on the predictors identified in the multivariate analysis, further probing was performed to investigate the cut-off values and odds ratios of these predictors for a high intraoperative blood loss. From available literature, there was no direct minimal clinically important difference (MCID) of intraoperative blood loss in AIS surgery. As total circulating blood volume accounts for approximately 7% of total body weight, this equals approximately 3500 ml in the average 50 kg patient [5]. Using the classification in hemorrhagic shock in the trauma setting [6], class 2 signified 15–30% blood volume loss, with tachycardia response. Therefore, we took 15% of total blood volume, which was about 500 ml, as the threshold for significant intraoperative blood loss. Cut-off values with optimal sensitivity and specificity (in percentages) of the predictive factors were derived from the Receiver Operating Characteristic (ROC) curve, whereas odds ratio of associating factors was obtained via logistic regression of the outcome of high intraoperative blood loss [7].

Data analysis was performed using the SPSS software version 24.0 (IBM®, Armonk, NY, US). *P*-value of < 0.05 was considered statistically significant. 95% CIs were reported where appropriate.

## Results

Two hundred and twelve patients were included in the review. The average age of the patients was 15.1 ± 2.7 years old (range 10–17 years old), and included 31 males and 181 females, with a mean BMI of 18.3 ± 2.8 kg/m^2^ (range 11.3–31.5 kg/m^2^). The preoperative hemoglobin was 12.2 ± 1.4 g/dL (range 9.3–16.4 g/dL). There were 142 (67%) type 1 curves, 41 (19.3%) type 3 curves, 25 (11.8) type 2 curves and 4 (1.9%) type 5 curves.

The mean duration of surgery was 280.4 ± 66.6 min (150.0–480.0 min). The most prevalent number of levels fused was 8 levels (27.8%, *n* = 58), ranging from 5 to 14 levels. The most prevalent number of anchor points was 10 (19.3%), ranging from 6 to 21 anchor points. The intraoperative mean blood loss per patient was 741.7 ± 403 ml (range 150–2200 ml) and the mean blood loss per level fused was 79.3 ± 37 ml (range 22–214 ml). Intraoperative blood transfusion was 409.5 ± 277.7 ml (range 0–1400 ml) of packed cells and 285.7 ± 250.9 ml (range 0–1000 ml) of fresh frozen plasma (FFP) per patient.

With regards to the type of instrumentation, pedicle screws were used in 76 (35.8%), pedicle screws and hooks in 108 (50.9%), only hooks in 9 (4.2%), pedicle screws with hooks and wires in 7 (3.3%) and pedicle screws with wires in 12 (5.7%) of the patients. Majority of the patients who had pedicle screw-hook systems had more that 80% of their anchor points as screws with a single hook at the top. None of the patients in the cohort had postoperative neurological deterioration.

The postoperative drain output from the spine wound was 254.6 ± 211.7 ml (range 5–840) and from the iliac wound was 178.4 ± 66.2 ml (range 65-410 ml). One hundred and five patients had an iliac wound drain while 107 patients did not have any iliac wound drain.

For wound complications, 147 (69.3%) patients had no wound healing complications (grade 0) in the spinal wound. 29 (13.7%) patients had grade I wound complications, 16 (7.5%) patients had grade II wound complications, 15 (7.1%) patients had grade III wound complications, 2 (0.9%) patients had grade IV wound complications and 3 (1.4%) patients had grade V wound complications. In the iliac wound, 191 (90.1%) patients had no wound healing complications (grade 0), 12 (5.7%) patients had grade I wound complications, 3 (1.4%) patients had grade II wound complications, 5 (2.4%) patients had grade III wound complications and 1 (0.5%) patient had grade IV wound complications.

Gender, preoperative hemoglobin level, preoperative Cobb angle, number of fused levels, operation duration and number of anchors were found to be significantly correlated with the amount of intraoperative blood loss (Table 1). A significant multivariate regression model was generated with these predictors. Significant predictors for intraoperative blood loss were identified as duration of operation (*r*_*s*_ = 0.65, *p* < 0.01), number of anchors (*r*_*s*_ = 0.47, *p* < 0.01) and male gender (*r*_pb_ = 0.3, *p* = 0.01) (Table 2). Intraoperative blood loss was found to be predictive by the following regression equation:

$$ amount\ of\ intraoperative\ blood\ loss\ (ml)=- 670.8+ 3.1\ast operation\ duration\ \left(\mathit{\min}\right)+ 33.8\ast number\ of\ anchors+ 158.9\ast gender\ \left( female: 1, male: 2\right).\left( Predicted\ model:F\ \left( 3, 208\right)= 66.486,p< 0.001, adjusted\ {R}^2= 0.482\right). $$

**Table 1:** Univariate analysis for factors associated with intraoperative blood loss

	Correlation Coefficient (95%CI)	*p*-value^a^
Age	−0.02 (− 0.15–0.12)	0.82
Gender^b^	0.30 (0.17–0.43)	< 0.01*
Body mass index	0.02 (− 0.12–0.16)	0.30
Smoking^b^	−0.07 (− 0.21–0.06)	0.28
Preoperative hemoglobin level	0.18 (0.04–0.31)	0.01*
Preoperative Cobb angle	0.16 (0.02–0.29)	0.02*
Number of fused levels	0.46 (0.34–0.58)	< 0.01*
Operation duration	0.65 (0.54–0.75)	< 0.01*
Preoperative albumin level	0.12 (− 0.01–0.26)	0.07
Number of anchors	0.47 (0.35–0.59)	< 0.01*

*CI* confidence interval

* Significance at *p*-value < 0.05

^a^ Spearman’s correlation test unless stated otherwise

^b^Point-serial correlation test

**Table 2:** Multivariate linear regression model for factors predicting intra-operative blood loss

Predicted model: F (3, 208) = 66.486, *p* < 0.001 *R* = 0.700, *R*^2^ = 0.490, Adjusted *R*^2^ = 0.482					
	Unstandardized Coefficients Β	Unstandardized Coefficients SE	Standardized Coefficients β	*p*-value	95% CI for β
Constant	−670.785	105.348		< 0.001*	− 878.47 to − 463.10
Operation duration (min)	3.131	0.331	0.518	< 0.001*	2.48 to 3.78
Number of anchors	33.840	7.631	0.241	< 0.001*	18.80 to 48.88
Gender (Female = 1; Male =2)	158.930	58.142	0.140	0.007*	44.31 to 273.55

*R* multiple correlation coefficient, *CI* confidence interval, *min* minute

* Significance at *p*-value < 0.05

**Fig. 2:**
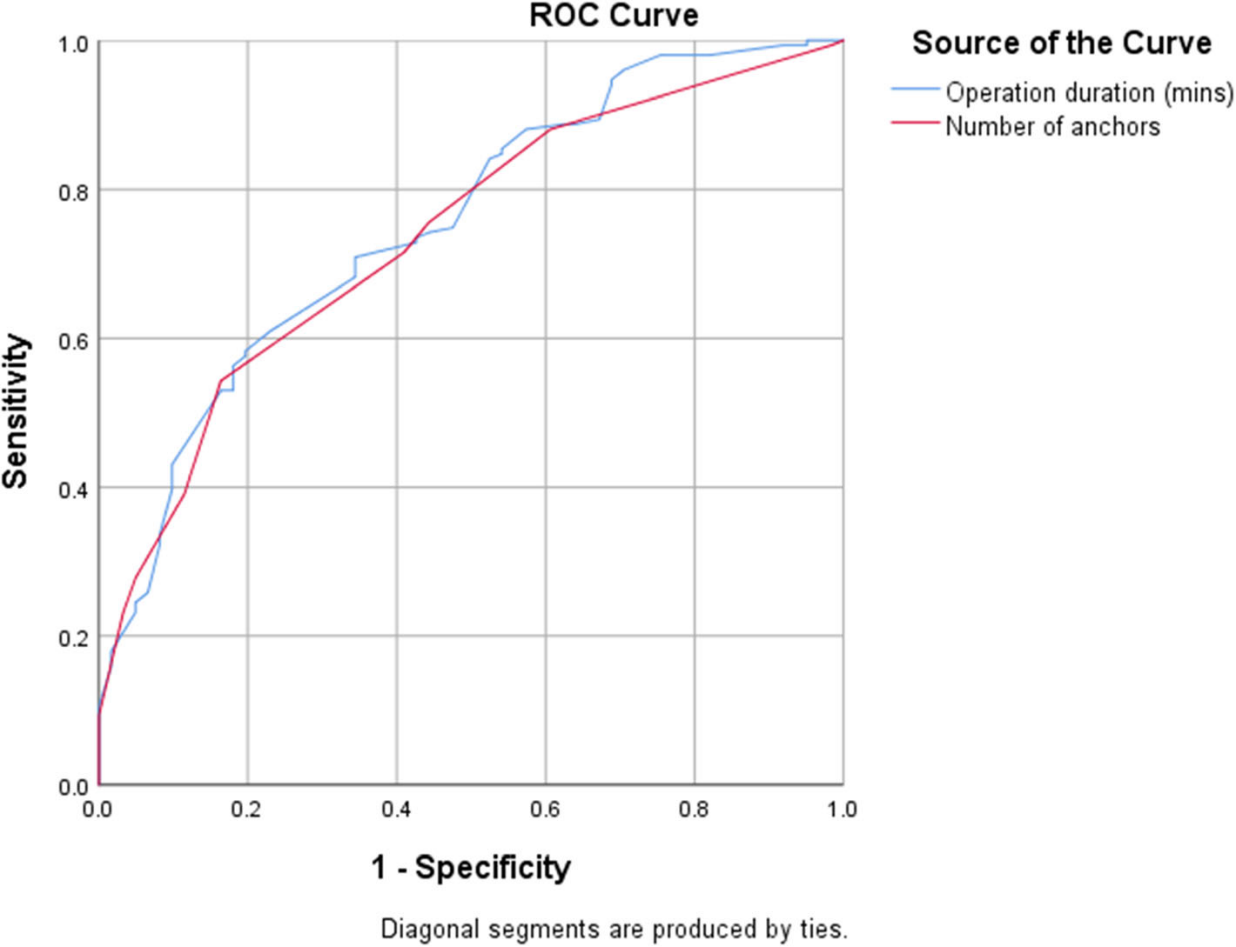
Receiver Operating Characteristic curve for predictive factors of high intraoperative blood loss (500 ml or greater)

The ROC analysis is shown in Fig. 2. An operation duration greater than 257.5 min (sensitivity: 70.9%, specificity: 65.6%, area under curve (AUC): 0.752, *p* < 0.01) and more than 10 anchors (sensitivity: 71.5%, specificity: 59.0%, AUC: 0.737, *p* < 0.01) predicted 500 ml or greater intraoperative blood loss. The adjusted odds ratio for significantly high blood loss (Table 3) was 1.015 (*p* < 0.001, 95% CI: 1.01–1.02) for operation duration and 1.364 (*p* = 0.005, 95% CI: 1.10–1.69) for the number of anchors used. Hence, for the increase of each minute of operation time, there was an increase of 1.5% higher odds of having high blood loss, and with one more anchor placed there was a 36.4% higher odds of having high blood loss.

**Table 3:** Multivariate logistic regression model for high intraoperative blood loss (≥ 500 mL)

Factors	Regression coefficient (B)	Standard error	Wald *X*^2^	*p*-value	Odds Ratio	95% CI
Constant	−7.610	2.927	6.759	0.009*		
Sex	−1.131	0.830	1.860	0.173	0.323	0.06 to 1.64
Preoperative Hb	0.195	0.142	1.893	0.169	1.216	0.92 to 1.61
Preoperative Cobb angle	0.020	0.021	0.897	0.344	1.020	0.98 to 1.06
Number of fused level	−0.162	0.133	1.473	0.225	0.851	0.66 to 1.10
Operation duration	0.015	0.004	14.931	< 0.001*	1.015	1.01 to 1.02
Number of anchors	0.310	0.110	8.013	0.005*	1.364	1.10 t0 1.69
Albumin	0.013	0.036	0.137	0.711	1.014	0.94 to 1.09

* Significance at *p*-value < 0.05

*CI* Confidence interval

## Discussion

Previous studies have attempted to study the predictive factors for blood loss in AIS surgery [8–10]. Our series is by far the group with largest sample size in posterior AIS surgery. Ialenti [10] studied 340 patients with AIS and found that males, preoperative kyphosis, and increased operative time were associated with increased intraoperative blood loss in posterior spinal fusion. However, when the patients included in their series were stratified into groups with different surgical approach, only 188 patients with AIS with posterior approach were studied. In our series, we found that increased number of anchors, male gender and increased operation duration were correlated with increased blood loss. The increased amount of blood loss in the male gender may be associated with a higher amount of muscle mass. Longer operative duration and higher number of anchors mean that the operation was more complex with longer time of dissection, which logically would have increased the amount of blood loss. Some of the predictive factors such as gender and number of anchors may not be modifiable. As a result, surgeons should try their best to minimize intraoperative blood loss such as involving dual attending surgeons.

In cases with significant intraoperative blood loss, blood transfusions of 2 to 6 units of blood may be required [11]. Modern transfusion is considered to be safe, but there still is a risk of infection with human immunodeficiency virus or hepatitis C [12], hemolytic (or other) transfusion reaction [13] and the development of graft versus host disease [14]. Massive transfusions can also cause metabolic issues including hypomagnesemia, hypochloremia, hyperkalemia, dilution of clotting factors and hypothermia [15–17]. Therefore, it is of paramount importance to minimize intraoperative blood loss, which in turn decreases the risk from blood transfusion.

The mean intraoperative blood loss per fused level of 79.3 ml in our series of patients and is lower than previously reported [8, 18, 19]. It is low even compared to those series that used hemostatic agents [18, 19]. It should be emphasized that the total blood loss in our patients also included blood loss from the iliac crest bone graft harvesting procedure. This leads us to believe that surgical technique including meticulous hemostasis is the main determinant of blood loss. It is also important to note that none of our patients used tranexamic acid during surgery. This assumption is strongly supported by the conclusions of Joanne Guay et al. [8] who found that there was no correlation between intraoperative bleeding and the Cobb angle, the mean arterial blood pressure, the central venous pressure, the quantity of epinephrine infiltrated, muscle relaxants or opioids used, or the minimal body temperature.

Autologous blood transfusion (ABT) can be made possible by preoperative autologous blood donation, intraoperative blood-saver and post-operative autologous blood transfusion drains. ABT could decrease the potential complications from allogenic blood transfusion, namely transfusion reactions, viral/prion disease transmission, coagulopathy, and the metabolic and immunological consequences of transfusing allogeneic blood [20]. Although preoperative autologous blood donation and intraoperative blood-saver had been shown to decrease the rate of allogenic blood transfusion [21], the authors still believe that minimizing intraoperative blood loss is important during operation. Precautions include hypotensive anesthesia, subperiosteal dissection, meticulous soft tissue haemostasis at all stages, and prevention of bone bleeding with bone wax after facetectomies are performed. There can be significant blood loss occurring during pedicle track creation and screw insertion. The following steps help to decrease blood loss:

1. Use bone wax or finger pressure to seal the track entry point between the steps of pedicle wall integrity checking, length measurement, tapping and screw insertion
2. Avoid the use of suction near the entry point or inserting the suction tip into the pedicle tract
3. Perform bloody steps such as decortication and bone cuts as late as possible and use bone wax for hemostasis
4. Perform the decortications and fusion process after all adjustments to the final constructs including intraoperative radiographs have been performed

None of the patients in this group had non-union as evidenced by implant failure, revision surgery or late loss of correction in the 2-year follow-up period.

Limitations of this study included its retrospective design though we have a standardised surgical approach. A multicenter study with a prospectively matched cohort that is randomized to different interventions can certainly draw a stronger conclusion. Nevertheless, we provide evidence in this study for factors that can be optimized to reduce intraoperative blood loss. Inferior articular processes excisions were performed in every fusion level in our operations, for the release and fusion. Ponte osteotomies and other types of osteotomies were not included, therefore prediction for intraoperative blood loss in AIS surgeries that involved osteotomies needs further study.

In conclusion, male gender, increased operation duration (≥257.5 min), and more anchors (≥10) predicted higher intraoperative blood loss in surgery for AIS.

## Data Availability

All data generated or analysed during this study are included in this published article.

## Abbreviations

AIS: adolescent idiopathic scoliosis
BMI: body mass index
